# Impaired branched chain amino acid oxidation contributes to cardiac insulin resistance in heart failure

**DOI:** 10.1186/s12933-019-0892-3

**Published:** 2019-07-05

**Authors:** Golam M. Uddin, Liyan Zhang, Saumya Shah, Arata Fukushima, Cory S. Wagg, Keshav Gopal, Rami Al Batran, Simran Pherwani, Kim L. Ho, Jamie Boisvenue, Qutuba G. Karwi, Tariq Altamimi, David S. Wishart, Jason R. B. Dyck, John R. Ussher, Gavin Y. Oudit, Gary D. Lopaschuk

**Affiliations:** 1 Cardiovascular Research Centre, University of Alberta, 423 Heritage Medical Research Centre, Edmonton, T6G 2S2 Canada; 2 Katz Centre for Pharmacy and Health Research, Faculty of Pharmacy and Pharmaceutical Sciences, University of Alberta, Edmonton, Canada; 3 Mazankowski Alberta Heart Institute, University of Alberta, Edmonton, Canada; 4 Alberta Diabetes Institute, University of Alberta, Edmonton, Canada; 5 Department of Biological Sciences, University of Alberta, Edmonton, Canada; 6 Metabolomics Innovation Centre, University of Alberta, Edmonton, Canada; 7 Divsion of Cardiology, Department of Medicine, University of Alberta, Edmonton, Canada; 8 Department of Pharmacology, College of Medicine, University of Diyala, Diyala, Iraq

**Keywords:** Branched chain amino acid, Heart failure, Insulin resistance, Dilated cardiomyopathy, Transverse aortic constriction

## Abstract

**Background:**

Branched chain amino acids (BCAA) can impair insulin signaling, and cardiac insulin resistance can occur in the failing heart. We, therefore, determined if cardiac BCAA accumulation occurs in patients with dilated cardiomyopathy (DCM), due to an impaired catabolism of BCAA, and if stimulating cardiac BCAA oxidation can improve cardiac function in mice with heart failure.

**Method:**

For human cohorts of DCM and control, both male and female patients of ages between 22 and 66 years were recruited with informed consent from University of Alberta hospital. Left ventricular biopsies were obtained at the time of transplantation. Control biopsies were obtained from non-transplanted donor hearts without heart disease history. To determine if stimulating BCAA catabolism could lessen the severity of heart failure, C57BL/6J mice subjected to a transverse aortic constriction (TAC) were treated between 1 to 4-week post-surgery with either vehicle or a stimulator of BCAA oxidation (BT2, 40 mg/kg/day).

**Result:**

Echocardiographic data showed a reduction in ejection fraction (54.3 ± 2.3 to 22.3 ± 2.2%) and an enhanced formation of cardiac fibrosis in DCM patients when compared to the control patients. Cardiac BCAA levels were dramatically elevated in left ventricular samples of patients with DCM. Hearts from DCM patients showed a blunted insulin signalling pathway, as indicated by an increase in P-IRS1ser636/639 and its upstream modulator P-p70S6K, but a decrease in its downstream modulators P-AKT ser473 and in P-GSK3β ser9. Cardiac BCAA oxidation in isolated working hearts was significantly enhanced by BT2, compared to vehicle, following either acute or chronic treatment. Treatment of TAC mice with BT2 significantly improved cardiac function in both sham and TAC mice (63.0 ± 1.8 and 56.9 ± 3.8% ejection fraction respectively). Furthermore, P-BCKDH and BCKDK expression was significantly decreased in the BT2 treated groups.

**Conclusion:**

We conclude that impaired cardiac BCAA catabolism and insulin signaling occur in human heart failure, while enhancing BCAA oxidation can improve cardiac function in the failing mouse heart.

**Electronic supplementary material:**

The online version of this article (10.1186/s12933-019-0892-3) contains supplementary material, which is available to authorized users.

## Background

A strong positive association between branched chain amino acids (BCAAs: valine, leucine and isoleucine) and whole-body insulin resistance has been demonstrated in obesity and diabetes [1–4]. Insulin resistance also occurs in the failing heart and contributes to the severity of cardiac dysfunction [5, 6]. However, while BCAAs increase in the failing heart [7, 8], it is not clear if this results in the development of cardiac insulin resistance and heart failure.

The mechanism by which BCAAs contribute to whole body insulin resistance has yet to be fully determined. One proposal is that high levels of BCAAs increase muscle BCAA oxidation, which decreases glucose and fatty acid oxidation, leading to insulin resistance [1]. However, we have shown in the heart that accumulation of BCAAs occurs in conjunction with a decrease, rather than an increase, in BCAA oxidation in obese mice [9]. In addition, the contribution of cardiac BCAA oxidation to cardiac energy production is less than 1%, suggesting that altered cardiac BCAA oxidation is unlikely to effectively compete with fatty acid and glucose oxidation as a source of acetyl CoA for the TCA cycle [9]. However, while altering BCAA oxidation is unlikely to directly compete with the use of other cardiac energy substrates, it still has the potential to alter cardiac BCAA levels. This is important as the accumulation of BCAAs can activate mammalian target of rapamycin (mTOR) [10] and impair insulin signaling, due to its ability to phosphorylate IRS1 via directly activating p70S6K through mTOR [1, 11–13]. Thus, a decrease in cardiac BCAA oxidation has the potential to increase cardiac BCAA levels, which may activate mTOR signaling and reduce cardiac insulin sensitivity. However, it remains unclear whether cardiac insulin signaling is impaired along with a decreased BCAA catabolism and activation of mTOR pathway in human failing hearts.

BCAA metabolism starts with transamination by branched chain aminotransferase (BCAT) to produce branched chain keto acids (BCKAs). BCKAs are then catabolized by branched-chain α-keto acid dehydrogenase (BCKDH) to produce substrates for the TCA cycle. BCKDH can be phosphorylated and inactivated by branched-chain α-keto acid dehydrogenase kinase (BCKDK) [14], or dephosphorylated and activated by a mitochondrial localized 2C-type serine-threonine protein phosphatase (PP2Cm) [15]. The first BCAA catabolic enzyme, BCATm has been shown to be reduced in human heart failure and TAC induced heart failure in mice [16]. Inhibition of BCATm should result in an accumulation of BCAAs and a decrease in BCKAs. Therefore, inhibition of BCATm would allow to increase BCAA levels and decrease BCKA levels. This may lead to a different scientific question and hypothesis. On the other hand, decreased BCKDH activity is the main cause of increased levels of BCAA and BCKA in maple syrup urine disease [17], and plays a role in mediating metabolic reprogramming in the failing hearts [2].

One potential approach to increasing BCAA oxidation is to inhibit BCKDK with 3,6-dichlorobenzothiophene-2-carboxylic acid (BT2) [2]. BT2 significantly reduces the phosphorylation of the BCKDH subunit E1α in the mouse heart, resulting in an increase in its activity [2]. However, whether BT2 actually enhances cardiac BCAA oxidation has not been directly assessed.

The objectives of this study were to determine whether cardiac BCAA catabolism is defective in patients with dilated cardiomyopathy (DCM), and whether this occurs in conjunction with an impaired cardiac insulin signaling along with an activation of the mTOR pathway. We also determined whether cardiac BCAA oxidation could be increased by BT2, and whether this improved cardiac function in the failing mouse heart.

## Methods

### Human explanted heart samples

Both male and female patients of ages between 22 and 66 years were recruited. The LV free wall samples were obtained at the time of transplantation (within 15 min post excision) from the patients with dilated cardiomyopathy (DCM, male n = 11; female n = 3; see Additional file 1: Table S1) and the non-failing control (NFC) hearts from non-transplanted donor hearts (NFC, male n = 5; female n = 2; median age = 47 year (range 31–56 year)) without heart disease, as previously described [18, 19]. Human echocardiographic data were obtained at the clinical pre-operative assessment, as described previously [18]. Patients were evaluated by standard echocardiographic methods to determine % ejection fraction (%EF) [20].

### Measurement of cardiac BCAAs

Metabolomic analysis of the human heart samples were performed to measure BCAA content, using an NMR spectroscopy. Frozen heart tissue samples (DCM, male n = 11; female n = 3) and non-transplanted donor hearts (NFC, male n = 5; female n = 2) were thawed on ice. An exact amount (0.5–0.75 g) of samples were transferred to a pre-chilled mortar and finely powdered in liquid nitrogen using pestle. 4 mL of cold methanol and 0.85 mL of cold water were added to homogenize the tissue sample for 3 min with a pestle. The extract was transferred to a 4 dram vial and 2.75 mL cold chloroform was added. The mortar was rinsed with 1.5 mL of cold methanol for the complete recovery of the extract. The vial was vortexed for 5 min and then centrifuged for 10 min at 3000 rpm. The supernatant was transferred into a new 4 dram glass vial and 2.75 mL cold chloroform and 4 mL cold water were added. Again vortexed vials for 3 min at high speed and centrifuged for 10 min at 3000 rpm. This gave a biphasic mixture. The upper aqueous layer (water-soluble metabolites) was transferred into a 15 mL falcon tube and 2.5 mL HPLC water was added to the water-soluble metabolite extract and flash freeze it in liquid nitrogen. The above falcon tube was lyophilized with frozen water-soluble metabolites for 24 h. 15 mg of the resultant freeze dried powder of water-soluble metabolites was then aliquoted for NMR analysis.

15 mg of lyophilized water-soluble extract from heart tissue was taken in 1.5 mL eppendorf tube. To this powder in eppendorf tube, 570 µL of water was added. The sample was sonicated for 15 min in a bath sonicator. 60 µL of reconstitution buffer (585 mM phosphate buffer with 11.67 mM DSS) and 70 µL of D_2_O were added. The solution was vortexed for 1 min and centrifuged at 10,000 rpm for 15 min at ambient temperature. Clear supernatant was transferred into NMR tube (Shigemi, Inc., Allison Park, PA) for NMR analysis.

All ^1^H-NMR spectra were collected on a Varian 500 MHz Inova spectrometer equipped with a 5 mm HCN Z-gradient pulsed-field gradient (PFG) cyrogenic probe (Varian Inc. Palo Alto, CA). ^1^H-NMR spectra were acquired at 25 °C using the first transient of the Varian tnnoesy pulse sequence, which was chosen for its high degree of selective water suppression and quantitative accuracy of resonances around the solvent. Water suppression pulses were calibrated to achieve a bandwidth of 80 gausses. Spectra were collected with 128 transient and 8 steady-state scans using a 4 s acquisition time (48,000 complex points) and a 1 s recycle delay.

Prior to spectral analysis, all FIDs were zero-filled to 64,000 data points and line broadened 0.5 Hz. The methyl singlet produced by a known quantity of DSS was used as an internal standard for chemical shift with reference set to 0 ppm and for quantification. All ^1^H-NMR spectra were processed and analyzed using the Chenomx NMR Suite Professional software package version 8.1 (Chenomx Inc., Edmonton, AB). The Chenomx NMR Suite software allows for qualitative and quantitative analysis of an NMR spectrum by manually fitting spectral signatures from an internal database to the spectrum. Typically, 90% of visible peaks were assigned to a compound and more than 90% of the spectral area could be routinely fit using the Chenomx spectral analysis software. Most of the visible peaks are annotated with a compound name.

### Histology

Masson’s trichrome staining and picrosirius red staining of paraffin-embedded left ventricular human heart sections taken mid-papillary were visualized using a Leica DMLA microscope (Leica Microsystems, Wetzlar, Germany) equipped with a Retiga 1300i FAST 1394 CCD camera (OImaging, Surrey, BC, Canada), as described previously [19]. Three representative images were taken from each sample and densitometric analysis was performed using the ImageJ software (National Institutes of Health, Bethesda, MD, USA).

### Animals

Mice were housed at the Health Sciences Lab Animal Services Facility at the University of Alberta in a temperature and humidity-controlled room with a 12 h light dark cycle. All the animals were euthanized in a non-fasted state at the end of experiments.

### Heart failure mouse model

Male C57BL/6 mice at 10 weeks of age were randomly assigned to either a sham (n = 24) or transverse aortic constriction (TAC, n = 26) surgical procedure. Mice were anesthetized with 0.75% isoflurane to surgical plane, following which a horizontal skin incision was made at the level of second intercostal space. A 6-0 silk suture was passed under the aortic arch followed by placing a bent 27-gauge needle next to the aortic arch. After constricting the aorta and closing the incision, the mice were allowed to recover on a warming pad. The sham animals went through the same procedure without constricting the aorta. Echocardiography was used to confirm a similar pressure gradient across the TAC.

### Treatment with the BCKDK inhibitor BT2

Sham (n = 12) and TAC mice (n = 13) were randomized into four groups, at 1-week post-surgery, for daily intraperitoneal injection (*IP*) for 3 weeks with either vehicle (5% DMSO, 10% cremophor EL, and 85% 0.1 M sodium bicarbonate, pH 9.0) or 6 mg/mL BT2 (3,6-dichloro-1-benzothiophene-2-carboxylic acid, Sigma-Aldrich) (final dose 40 mg/kg/day).

### Assessment of BCAA oxidation

The isolated working heart perfusion principle and experimental procedure were followed as described previously [21]. Isolated working hearts were perfused with Krebs–Henseleit solution (118.5 mM NaCl, 25 mM NaHCO_3_, 1.2 mM MgSO_4_, 4.7 mM KCl, 1.2 mM KH_2_PO_4_, 2.5 mM CaCl_2_) supplemented with 0.8 mM palmitate bound to 3% fatty acid free bovine serum albumin, 5 mM glucose, 0.15 mM leucine, 0.15 mM isoleucine, and 0.2 mM valine. BCAA oxidation was measured by trapping and measuring ^14^CO_2_ released by the metabolism of [U-^14^C] valine/leucine/isoleucine. The ^14^CO_2_ released during BCAA oxidation (respective keto acid decarboxylation and tricarboxylic acid cycle) was trapped using 1 M hyamine hydroxide. Quantitative collection of 14CO_2_ was performed by continuously bubbling the outflow air from the perfusion apparatus through 15 mL of hyamine hydroxide and then, sampling the hyamine hydroxide (300 µL) every 10 min, starting at 0 min. The hyamine hydroxide samples were counted using CytoScint scintillation cocktail. Quantitative 14CO_2_ production was measured by adding together the values for 14CO_2_ obtained from the outflow air and solution. However, there is a limitation of measuring the BCAA oxidation in this method. BT2 (200 µM) was also added to the perfusate when assessing the acute BT2 effects on BCAA oxidation. At the end of the 60 min perfusion period, hearts were snap frozen in liquid N_2_ immediately after perfusion and stored at − 80 °C for later biochemical analysis.

### Mice echocardiography and tissue doppler imaging

Echocardiographic analysis on mice was performed at 0-week (baseline) and at 4-weeks post-surgery, by using a Visualsonic Vevo 770 high-resolution echocardiography imaging system equipped with a 30-MHz transducer (RMV-707B; VisualSonics, Toronto, Canada). M-mode images were obtained for measurements of ejection fraction (%EF), LVPW;d, LVPWD;s, and LV mass. A non-invasive measurement of area under the curve (mmHg) in pulse wave Doppler-mode was used to measure the pressure gradient across the transverse aortic constriction site.

### Western blot analysis

Frozen heart tissues were homogenized in a buffer containing 50 mM Tris HCl, 1 mM EDTA, 10% glycerol, 0.02% Brij-35, 1 mM DTT, protease and phosphatase inhibitors (Sigma). Thirty µg of protein from the resulting supernatant were subjected to SDS-PAGE followed by western blotting procedures. Primary antibodies include BCKDH (Ab138460), BCKDK (Ab128935) and mitochondrial protein phosphatase 2C (PP2Cm: Ab135286) from Abcam; p-p70s6kinase Thr389 (9206s), p70S6kinase (9202s), p-mTOR Ser2448 (2971s), mTOR (4517s), pAkt Ser473 (9271s), AKT (9272s) and pGSK3 α/β Ser21/9 (9331s), GSK (9315s), pIRS1 Ser636/639 (2388s) and IRS1 (2382s) from Cell signaling; α tubulin (Sigma, T6074); pBCKDH Ser293 (Bethyl A303-567A); and mitochondrial BCAT(BCATm, Thermo Scientific, PA5-21549). Enhanced chemiluminescence (Perkin Elmer) was used to visualize protein bands on autoradiography films, and quantification of the protein bands was performed with Image J.

### Statistical analysis

Data are presented as the mean ± SEM. P value of < 0.05 were considered statistically different. Data from the human studies were analyzed by an unpaired Student’s *t* test. In the animal studies with > 2 groups, ANOVA or appropriate nonparametric tests were applied to analyze the difference.

## Results

### Patients with DCM have a markedly decreased %EF, decreased cardiac SERCA2 expression, increased cardiac α-SKA, and increased cardiac fibrosis

**Fig. 1:**
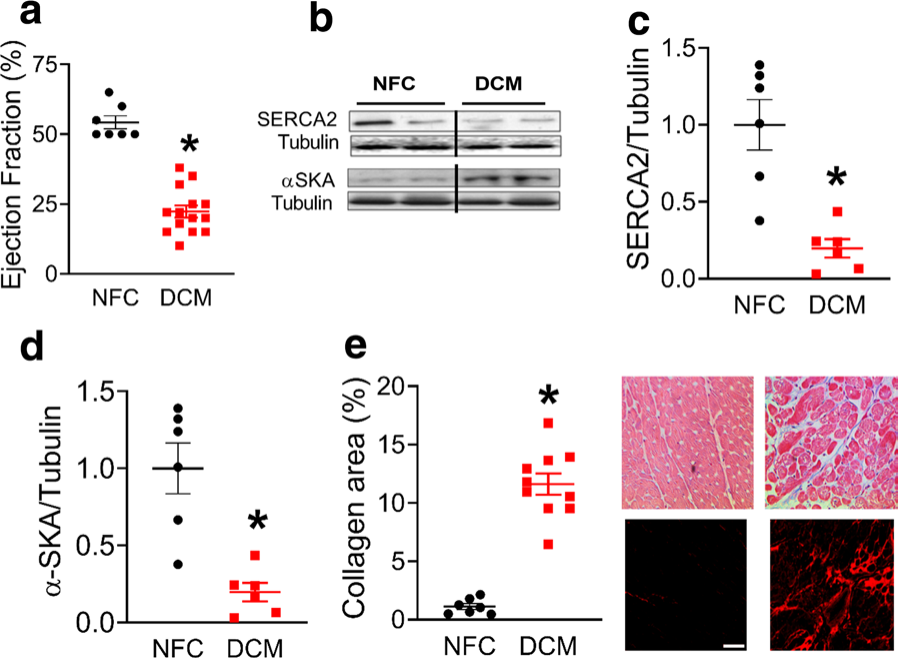
Decreased %EF, SERCA2 expression and increased α-SKA and fibrosis in patients with DCM. Ejection fraction (%) (NFC, n = 7; DCM, n = 14) based on echocardiographic analysis at the clinical pre-operative assessment (**a**). Representative blots of SERCA2 and α-SkA with Tubulin as the loading control (**b**). Densitometry analysis of SERCA2 (**c**) and α-SkA (**d**), normalized to Tubulin, respectively (NFC, n = 6; DCM, n = 6). Collagen visualization and trichrome staining expressed as collagen area (%) (NFC, n = 7; DCM, n = 10) (**e**). Data are presented as mean ± SEM. Data were analysed by t-test. *p < 0.05 was considered statistically significant

To characterize the cardiac dysfunction in patients with DCM, echocardiographic analysis at the clinical pre-operative assessment were performed. The patients with DCM had significantly reduced %EF, a representative parameter of systolic dysfunction, compared to the non-failing control (NFC) (p < 0.05, Fig. 1a). At a molecular level, a decrease in expression of SERCA2 (Fig. 1b, c) was observed in the DCM hearts, while an increase in alpha skeletal muscle actin (α-SKA) expression was seen (Fig. 1b, d). In addition, accumulation of collagen (Fig. 1e), as a pathologic feature of fibrosis, was evident in the DCM hearts relative to the NFC hearts.

### Patients with DCM have elevated levels of cardiac BCAA and an impaired BCAA catabolic pathway

**Fig. 2:**
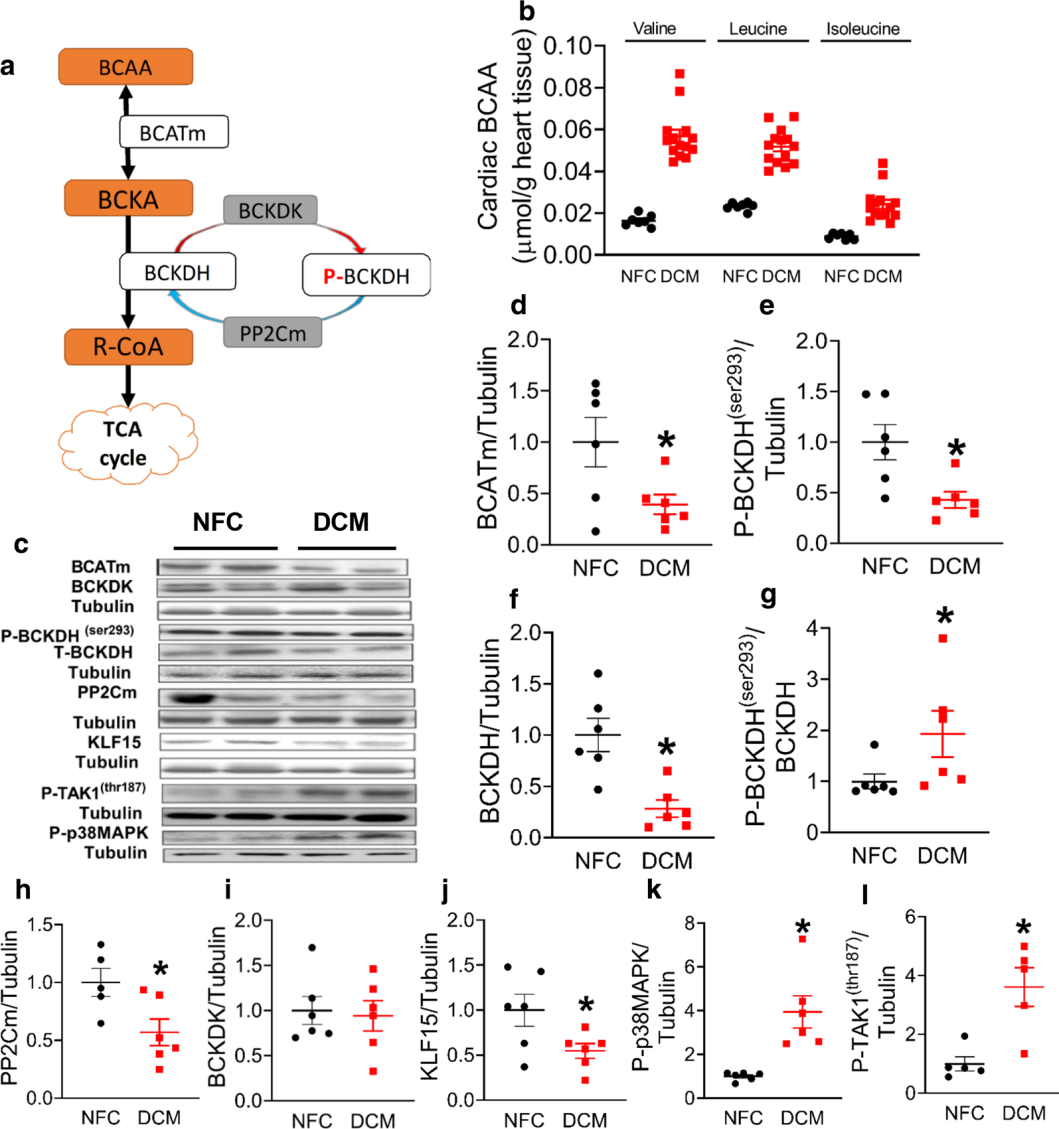
Elevated BCAAs and impaired BCAA catabolic pathway in the hearts of patients with DCM. Schematic drawing of BCAA catabolic pathway (**a**). Levels of cardiac BCAAs (NFC, n = 7; DCM, n = 14) (**b**). Representative blots of proteins of the BCAA catabolic pathway (**c**). Densitometry analysis of BCATm normalized to Tubulin (**d**). Densitometry analysis of P-BCKDH (**e**), and BCKDH (**f**), normalized to Tubulin, respectively. Ratio of P-BCKDH ^(Ser 293)^ over the total BCKDH (**g**). Densitometry analysis of PP2Cm (**h**), BCKDK (**i**) and KLF15 (**j**), normalized to Tubulin, respectively. Densitometry analysis of P-p38MAPK (**k**) and P-TAK1 ^(thr187)^ (**l**), normalized to Tubulin, respectively (n = 6/group). Data are presented as mean ± SEM. Data were analysed by t-test. *p < 0.05 was considered statistically significant

To explore the potential association between heart failure and BCAA, cardiac BCAA levels and the key enzymes for BCAA catabolism were assessed in failing human explanted hearts with DCM (Fig. 2a). Elevated cardiac BCAA levels were observed in the DCM hearts (Fig. 2b), in conjunction with a reduced protein expression of mitochondrial BCATm (Fig. 2c, d). In addition, a significant reduction in the total expression of BCKDH (Fig. 2c, f) and phosphorylation of BCKDH was seen in the DCM hearts (Fig. 2c, e). Decreased protein expression could be the result of reduced phosphorylation of BCKDH in DCM patients, which was observed when normalised to the loading control (Fig. 2e). As a result, an increased ratio of P-BCKDH over the total BCKDH was observed in the DCM hearts relative to the NFC hearts (Fig. 2g), implying a decreased BCKDH activity in the DCM hearts that is controlled at both translational and post-translational levels. Of interest, expression of cardiac PP2Cm, the phosphatase responsible for dephosphorylating and activating BCKDH, was reduced in the DCM hearts (Fig. 2h) without an alteration in BCKDK expression (Fig. 2i). Furthermore, a reduced protein expression of cardiac KLF15, an upstream modulator of BCAA catabolic enzymes [2, 22, 23], was evident in the DCM hearts (Fig. 2j). This suggests an association between the accumulation of cardiac BCAA and KLF15 mediated down-regulation of BCATm and PP2Cm.

Transforming growth factor-β mediated activation of p38MAPK is necessary and sufficient to inhibit KLF15 expression [24]. Indeed, the phosphorylation of p38MAPK (Fig. 2c, k) was increased in the DCM hearts as was the phosphorylation of transforming growth factor-β activating kinase 1 (P-TAK1) (Fig. 2c, l), suggesting that accumulation of cardiac BCAA in the DCM hearts may be triggered by the activation of TAK1, thereby inhibiting KLF15 to down-regulate BCAA catabolism.

### Patients with DCM have impaired cardiac insulin signaling and activation of the mTOR pathway

**Fig. 3:**
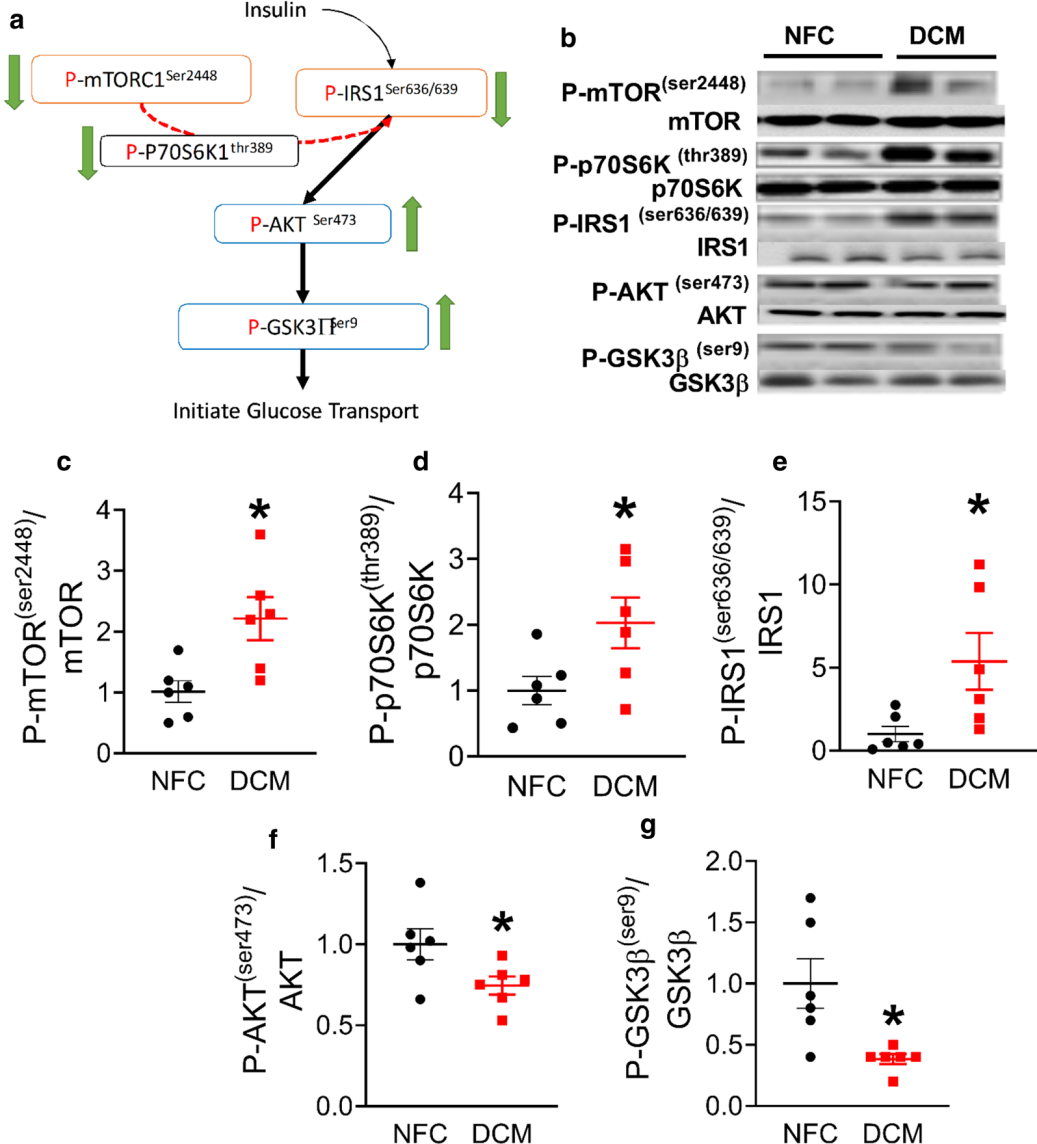
Impaired insulin signaling and activation of mTOR pathway in the hearts of patients with DCM. Schematic drawing of mTOR activation and insulin signaling under normal condition (**a**) and representative blots for proteins in the signaling pathway (**b**). Densitometry analysis of mTOR ^(Ser2448)^ (**c**) and P-p70S6K^(thr389)^ (**d**), P-IRS1^(Ser636/639)^ (**e**), P-AKT^(Ser473)^ (**f**) and P-GSK3ß^(Ser9)^ (**g**), normalized to its total protein, (n = 6/group). Data are presented as mean ± SEM. Data were analysed by t-test. *p < 0.05 was considered statistically significant

To understand if activation of mTOR occurred along with an impaired insulin signaling in the DCM hearts (Fig. 3a), the mTOR and insulin signaling pathway was examined. An increase in the phosphorylation of mTOR1^Ser2448^ was observed in the DCM hearts (Fig. 3b, c), in conjunction with an elevated phosphorylation of p70S6K (Fig. 3b, d) and P-IRS1^ser636/639^ (Fig. 3b, e), the down-stream targets of mTOR. In addition, P-AKT (Fig. 3b, f), a down-stream target of IRS1, was decreased in the DCM hearts, as was P-GSK3 (Fig. 3b, g), a down-stream target of P-AKT. Thus, impaired insulin signaling co-exists with an activated mTOR pathway in human DCM hearts.

### Treatment of BT2 increases cardiac BCAA oxidation in mouse hearts

**Fig. 4:**
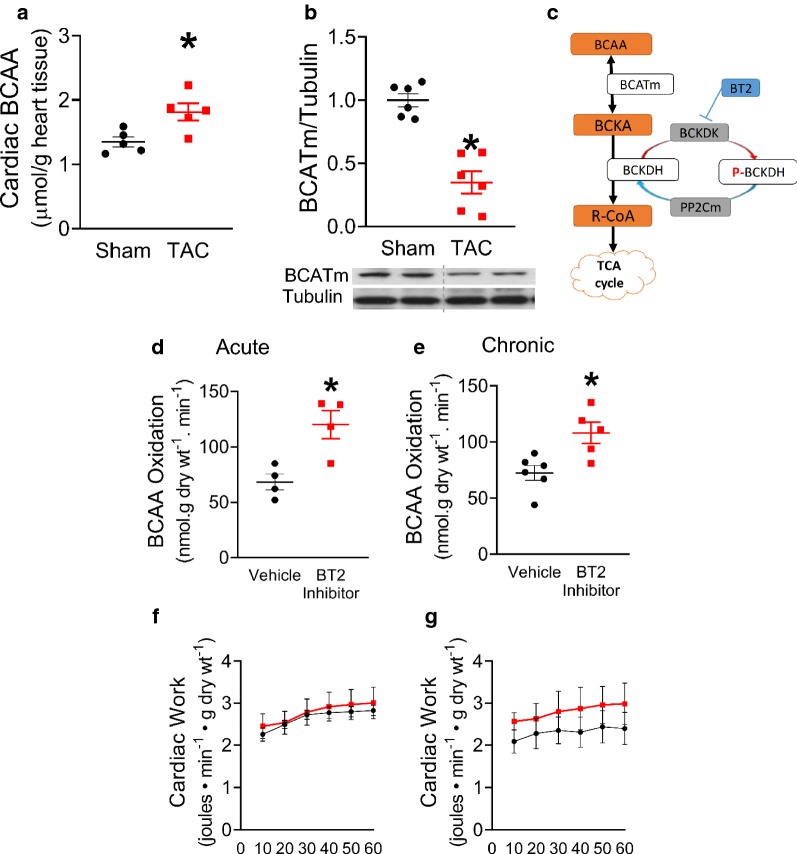
Accumulation of cardiac BCAAs in TAC mouse hearts. BT2 increases cardiac BCAA oxidation in mouse. Levels of cardiac BCAAs (n = 5/group) (**a**). Densitometry analysis of BCATm protein expression normalized to Tubulin (n = 6/group) (**b**). Schematic drawing of BT2 inhibition on BCAA catabolic pathway (**c**). Rates of BCAA oxidation in normal hearts perfused with BT2 (200 µM) (n = 4/group) (**d**). Rates of BCAA oxidation in normal hearts at 3 week post daily BT2 treatment (40 mg/kg/day) (Vehicle, n = 6; BT2 Inhibitor, n =) (**e**). Cardiac work for acute and chronic BT2 treatment was measured as a functional parameter during ex vivo working heart perfusion **f** and **g** respectively. Data are presented as mean ± SEM. Data were analysed by t-test. *p < 0.05 was considered statistically significant

Similar to the human DCM hearts, accumulation of BCAA was also evident in the mouse failing heart induced by TAC (Fig. 4a). Interestingly, levels of cardiac BCAAs are much higher in mouse compared to human samples, which is consistent with the finding previously published by Sun et al. [16]. Accumulation of BCAAs are concomitant with a reduced protein expression of BCATm (Fig. 4b). Inhibiting BCKDK activity by BT2 (Fig. 4c) can effectively activate BCKDH in various tissues [25]. Therefore, we explored whether the effect of BT2 could be translated into enhanced BCAA oxidation in the mouse heart. The acute effect of BT2 was examined in isolated working mouse hearts. BCAA oxidation was significantly increased in the mouse heart with BT2 relative to vehicle (Fig. 4d). Similarly, a chronic treatment with BT2 via an *IP* injection for 3 week enhanced cardiac BCAA oxidation in mice (Fig. 4e). Either, acute or chronic treatment of BT2, did not cause any changes in the ex vivo working heart function, as observed in cardiac work (Fig. 4f, g) respectively.

### Stimulating BCAA oxidation improves  % ejection fraction (%EF) without altering cardiac hypertrophy

**Fig. 5:**
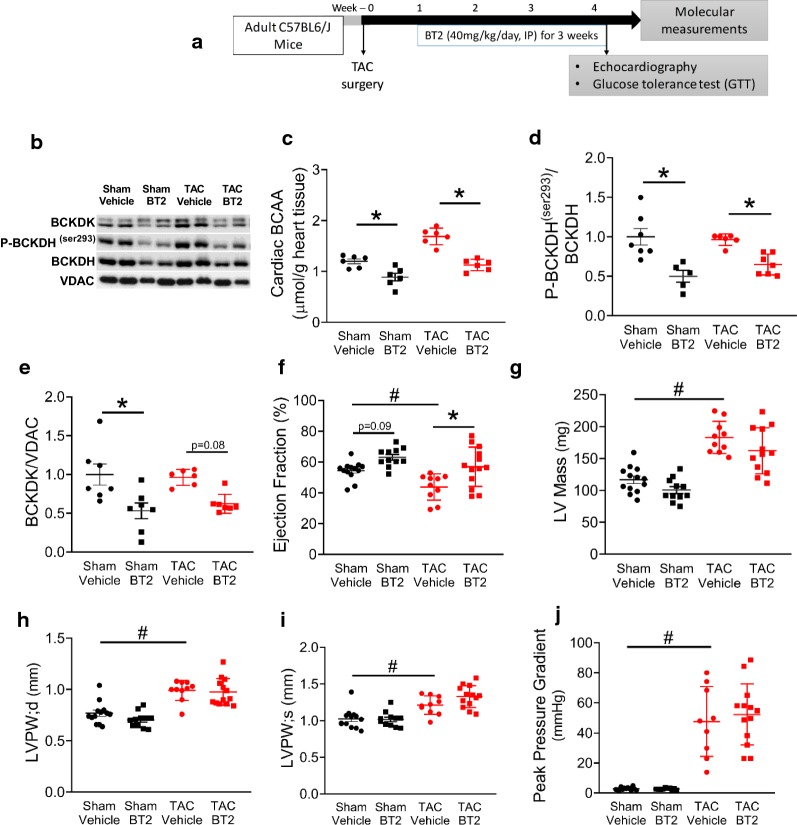
Improved %EF and unaltered cardiac hypertrophy in TAC mouse hearts post chronic treatment with BT2. Schematic drawing of the protocol for TAC surgery along with BT2 treatment (40 mg/kg/day), a GTT was conducted followed by 6 h fasting (2 g/kg body weight) (**a**). Representative blots of P-BCKDH, total BCKDH and BCKDH with VDAC as a loading control (**b**). Levels of cardiac BCAAs (n = 6/group) (**c**). Densitometry analysis of P-BCKDH normalized to total BCKDH (**d**), and BCKDK normalized to VDAC (n = 6/group) (**e**). Changes in ejection fraction (%) (**f**). Changes in left ventricular mass (**g**), and posterior wall thickness in diastole (**h**) and systole (**i**), as well as peak pressure gradient (**j**) at 4 week post daily BT2 treatment (n = 9–14/group). Data are presented as mean ± SEM. Data were analysed by using one-way ANOVA with multiple comparison. * or #, as p < 0.05 was considered statistically significant

Since we demonstrated that BT2 could increase cardiac BCAA oxidation (Fig. 4), we explored if chronic treatment of BT2 could improve cardiac function in failing mouse hearts (Fig. 5a). There was no change observed in whole body glucose tolerance test between the groups (data not shown). BT2 treatment significantly reduced BCAA accumulation in both sham and TAC hearts (Fig. 5c). As expected, BT2 treatment effectively reduced cardiac P-BCKDH (Fig. 5b, d), along with a reduction on cardiac BCKDK expression (Fig. 5b, e) in both sham and TAC mouse hearts. In addition, the TAC mouse hearts showed an impaired %EF when compared to sham mice (Fig. 5f). Treatment of TAC mice with BT2 significantly increased %EF in the TAC hearts. BT2 also increased in %EF in the sham hearts, but this increase did not reach the statistical significance (p = 0.09) (Fig. 5e). Despite of an increase in %EF, treatment of BT2 had no effect on altering cardiac hypertrophy in the TAC mice, as reflected by the unaltered LV mass (Fig. 5g), as well as the lack of change in posterior wall thickness in diastole (Fig. 5h) and in systole (Fig. 5i). To rule out the possibility that the increase in the %EF could be due to the differences in the severity of heart failure among the TAC hearts, we also examined the pressure gradient across the aortic banding. As expected, the pressure gradients were greater in the TAC mice than in sham mice (Fig. 5j), but were not altered by the treatment of BT2, confirming that the effect of BT2 on increasing cardiac %EF was not due to a difference in the severity of the TAC.

## Discussion

There are several novel findings of this study: (1) while a number of human studies have predicted a relationship between circulating BCAAs and insulin resistance [1, 3, 4, 26, 27], this is the first study to demonstrate that accumulation of cardiac BCAAs (with a coordinated decrease in BCAA catabolic enzymes) is associated with impaired cardiac insulin signaling in the failing human heart, (2) impaired insulin signaling is accompanied by activation of the mTOR pathway in the human failing heart, (3) reduction of KLF15 expression is associated with the impaired BCAA catabolism in the human failing hearts through a mechanism that may be associated with the activation of TAK1 and p38MAPK, and (4) stimulation of BCAA oxidation in the failing mouse heart improves contractile function. These findings support the concept that increasing cardiac BCAA oxidation may be a potential therapeutic strategy to treat heart failure.

A hypothesized mechanism of insulin resistance has linked increased BCAA or BCKA levels to the activation of mTOR signaling [28–30]. Our previous studies have shown, by directly measuring BCAA oxidation in hearts of mice subjected to a high-fat diet, that rates of BCAA oxidation are decreased, rather than increased, in insulin-resistant hearts [9]. In the current study, decreased cardiac BCAA oxidation, as evidenced by decreased expression of BCAA catabolic enzymes, was also observed in heart failure patients. In line with this, it has been demonstrated that defects in BCAA oxidation enzymes in diseases, such as methylmalonic acidemia are associated with human cardiomyopathy [29]. These results suggest that a rise in BCAA and/or BCAA metabolites is attributable to a decline in cardiac BCAA oxidation that results in the development of cardiac insulin resistance. Accumulation of BCKA levels with unaltered BCAA levels has been reported in heart failure patients [2]. However, in contrast, we observed a marked increase in BCAA levels in our human failing heart samples (Fig. 2). Elevated levels of intracellular BCAA has been observed in cultured cardiomyocytes coinciding with an activation of mTOR [31], which is consistent with the observations not only in our patients with DCM hearts, but also in murine failing hearts induced by either pressure overload or myocardial infarction [2, 25, 32, 33]. In our study, we did not assess the levels of cardiac BCKA in the DCM hearts, neither the causal effect of BCAAs or its metabolites on mTOR activation. Thus, the possibility that mTOR was activated as a result of increased autophagy in the heart failure can’t be rule out [34]. As a result, their potential contribution to the activated mTOR pathway and impaired insulin signaling remains unclear.

Of interest, an experiment conducted in mice with BCATm knockdown indicated that BCKA must be converted back to BCAA for insulin resistance to occur [35]. Thus, the question as to whether the accumulation of BCKA rather of BCAA is a critical factor responsible for metabolic consequences needs further clarification. A pharmacological or a transcriptional inhibition of the BCATm should result in accumulation of the BCAAs and reduce its oxidation, also, this should indicate if BCAAs are critical factor responsible for insulin resistance in the heart failure. Furthermore, there is a very little information available about the cytosolic and/or mitochondrial BCAA accumulation, as well as, its catabolic enzymes distribution throughout the cell and tissue. Based on the concept that in the BCAA catabolic pathway, BCAAs are first converted into branched-chain alpha-ketoacids (BCKA) by BCAT in a reversible reaction, it is possible that accumulation of mitochondrial BCAA could export back to cytosol. However, the cytosolic BCAT is not primarily expressed in the heart, but rather in the brain, testes and ovaries. This suggests that the backward reaction from BCKA to BCAA is unlikely a major pathway in the heart. In addition, they are the most hydrophobic [36], which suggests that they are not freely exported back into cytosol.

Despite evidence for a role for KLF15 in regulating cardiac BCAA catabolic gene expression [37–40], the information regarding the regulatory molecules of KLF15 is scarce. It has been reported that stimulation of transforming growth factor beta (TGFβ) expression in myocytes can activate p38α kinase (p38MAPK) via TGFβ-activated kinase 1 (TAK1) and cause inhibition of KLF15 expression [22]. Of interest, we found an increased phosphorylation of TAK1 and p38MAPK, along with a decrease in KLF15 expression in the human DCM hearts. These results suggest that TGFβ mediated TAK1/p38MAPK/KLF15 signaling may be a mechanism underlying the defect of BCAA catabolism in the human DCM hearts. This notion is further supported by a study showing that increased production of TGFβ can cause cardiomyocyte hypertrophy along with a p38MAPK-dependent suppression of KLF15 mRNA and protein [41]. In addition, it has recently been demonstrated that the cAMP response element binding protein (CREB) contains a binding element on the KLF15 promoter, and overexpression of CREB is sufficient to attenuate high glucose induced downregulation of KLF15 and BCAA catabolic enzymes [31]. As p38MAPK is an upstream modulator of CREB in rat hearts [42], it is reasonable to propose that signaling through the TAK1/p38MAPK/CREB/KLF15 axis may be a mechanism responsible for mediating BCAA catabolism in the heart.

The beneficial effects of the BT2 inhibitor have been demonstrated by others in the failing mouse heart, due to inhibition of BCKDK activity and a decreased phosphorylation of BCKDH [2]. In mice hearts, we show that BT2 can significantly enhance BCAA oxidation, presumably secondary to activation of BCKDH (Fig. 4). We and others have shown that the contribution of cardiac BCAA oxidation to energy production is as low as 1–3% [9, 43]. Therefore, increasing BCAA oxidation cannot effectively compete with fatty acid and glucose oxidation as a source of acetyl CoA for the TCA cycle. Instead, we propose that the BT2-mediated rise in cardiac BCAA oxidation in the failing hearts would decrease BCAA levels, and therefore improve cardiac insulin sensitivity. Importantly, we have also demonstrated that the pressure gradient across the aortic banding is greater in the TAC hearts relative to sham, while BT2 has no effect on changing it. Thus, the improved %EF in the TAC mouse heart is more likely due to the effect of BT2 on increasing BCAA oxidation, rather than a surgical effect on manipulating the severity of heart failure.

## Limitations

(1) Activation of the signaling through the TAK1/p38MAP/KLF15 axis could be a potential mechanism associated with the impaired BCAA catabolism in human DCM hearts. However, further studies with the cell culture system and relative animal models are needed to corroborate this conclusion; (2) The effect of BT2 on altering cardiac BCAA content along with enhancing cardiac BCAA oxidation and ex vivo cardiac work needs to be investigated in mouse TAC hearts, which would further support the improvement of %EF in vivo. Also, level of fibrosis in this animal could be one of the explanation of this improvement; (3) In this study, some variations in the expression of the BCAA catabolic enzymes in the failing heart occurred between mice versus humans. However, there also are similarities. For instance, in this current study, we found a reduction of the upstream enzyme BCATm and an accumulation of the cardiac BCAAs due to TAC surgery in mice, a finding also seen in the human failing heart samples. Similar to this finding, in a previous study we demonstrated that cardiac insulin resistance due to high fat diet-induced obesity in mice is associated with a reduced BCAA oxidation, but we did not see any changes in P-BCKDH and BCKDH levels [9]. Unlike what we found in our study, a reduction of BCKDH complexes due to TAC surgery in mice has been reported by Sun et al. [16], which parallels what we see in the human heart failure studies. Therefore, while a number of changes in BCAA catabolism were similar between mouse and human failing hearts, other changes were not similar. Some of these differences may be related to the severity of heart failure between the mice and the humans. The severity of the heart failure due to the TAC surgery did not reach to the level as we have anticipated. Although we have seen a significant change in upstream BCAA catabolic enzymes and cardiac BCAAs, however, no significant changes in the downstream enzymes or insulin signaling markers may be a result of the less severe TAC surgery.

## Conclusions

In conclusion, signaling through the TAK1/P38MAPK axis in the human failing heart leads to an inhibition of KLF15 expression and inhibition of the BCAA catabolic pathway, resulting in elevated cardiac BCAA levels. The result of this is a BCAA mediated activation of the mTOR initiated P-p70S6K/P-IRS1^ser636^ pathway, which results in blunted insulin signaling. Lowering cardiac BCAA levels (or BCAA metabolites) by enhancing BCAA oxidation, may have therapeutic potential for treating heart failure.

## Additional file

**Additional file 1: Table S1.** Clinical profile of patients with dilated cardiomyopathy.

## Data Availability

All data and materials used in the current study are available from the correspondent authors upon request.

## Abbreviations

Akt: protein kinase A
BCAA: branched chain amino acids
BCATm: mitochondrial branched chain aminotransferase
IRS-1: insulin receptor substrate 1
KLF15: Krüppel-like factor 15
p38 MAPK: p38 mitogen-activated protein kinases
TAK1: transforming growth factor beta-activated kinase 1
TCA: tricarboxylic acid
TAC: transvers aortic constriction
DCM: dilated cardiomyopathy
NFC: non failing hearts
PP2Cm: 2C-type serine-threonine protein phosphatase
BCKDH: branched-chain α-keto acid dehydrogenase
BCKDK: branched-chain α-keto acid dehydrogenase kinase
