# Supplementary material for: Impaired branched chain amino acid oxidation contributes to cardiac insulin resistance in heart failure
Source: Cardiovasc Diabetol. 2019 Jul 5;18:86. doi: 10.1186/s12933-019-0892-3 (PMC6610921; doi:10.1186/s12933-019-0892-3)
Supplement: Supplementary file 1 — Additional file 1: Table S1. Clinical profile of patients with dilated cardiomyopathy. [file 12933_2019_892_MOESM1_ESM.docx]

**Table S1. Clinical profile of patients with dilated cardiomyopathy.**

| n | 14 |
| --- | --- |
| Age at transplant (years) | 52 (42-61) |
| Sex (Male/Female) | 11/3 |
| Disease duration (months) | 64 (7.8-98) |
| **Comorbidities** |  |
| Liver disease | 1/14 |
| Kidney disease | 4/14 |
| COPD | 1/14 |
| Thyroid | 2/14 |
| Diabetes Mellitus | 1/14 |
| **Medications** |  |
| ACEI/ARB | 13/14 |
| β blocker | 12/14 |
| Diuretic | 14/14 |
| Anti-platelet | 2/14 |
| Statin | 1/14 |
| **Echocardiography** |  |
| LVESD (cm) | 5.08±0.31 |
| LVEDD (cm) | 6.15±0.37 |

COPD, chronic obstructive pulmonary disease; ACEi, angiotensin-converting enzyme inhibitor; ARB, angiotensin receptor blocker; LVEDD=LV end-diastolic dimension; LVESD=LV end-systolic dimension.
